# Concurrent initiation of intra-aortic balloon pumping with extracorporeal membrane oxygenation reduced in-hospital mortality in postcardiotomy cardiogenic shock

**DOI:** 10.1186/s13613-019-0496-9

**Published:** 2019-01-23

**Authors:** Kai Chen, Jianfeng Hou, Hanwei Tang, Shengshou Hu

**Affiliations:** State Key Laboratory of Cardiovascular Disease, Fuwai Hospital, National Center for Cardiovascular Diseases, Peking Union Medical College, Chinese Academy of Medical Sciences, 167A Beilishi Road, Xi Cheng District, Beijing, 100037 China

**Keywords:** Cardiac surgery, Postcardiotomy cardiogenic shock (PCS), Extracorporeal membrane oxygenation (ECMO), Intra-aortic balloon pumping (IABP), Concurrent initiation, Outcomes, Risk factors

## Abstract

**Background:**

Veno-arterial extracorporeal membrane oxygenation (VA-ECMO) is widely used in postcardiotomy cardiac shock (PCS). The factors that affect mortality in patients who receive ECMO for PCS remain unclear. In this study, we analyzed the outcomes, predictive factors and complications of ECMO use for PCS.

**Methods:**

A total of 152 adult subjects who received VA-ECMO for PCS in Fuwai Hospital were consecutively included. We retrospectively collected the baseline characteristics, outcomes and complications. Baseline characteristics were compared between survivors with non-survivors, and logistic regression was performed to identify predictive factors for in-hospital mortality.

**Results:**

The mean age of the subjects was 49.5 ± 14.1 years, with a male dominancy of 73.7%. The main surgical procedures were heart transplantation (32.2%), coronary artery bypass graft (17%) and valvular surgery (11.8%). Intra-aortic balloon pumping (IABP) was initiated concurrently with ECMO in 32.2% subjects and sequentially in 18.4% subjects. The ECMO weaning rate was 56.6%, and the in-hospital mortality was 52.0%. When compared with non-survivors, survivors had less hypertension (15.1% vs. 35.4%, *p *= 0.004), secondary thoracotomy before ECMO initiation (19.2% vs. 39.2%, *p *= 0.007), pre-ECMO cardiac arrest/ventricular fibrillation (11.0% vs. 34.2%, *p *= 0.001), bedside implantation of ECMO (11.0% vs. 41.8%, *p *< 0.001), and more transplant procedure (45.2% vs. 20.3%, *p *= 0.001), concurrent IABP initiation with ECMO (41.1% vs. 24.1%, *p *= 0.025). Multivariate logistic regression indicated concurrent IABP initiation with ECMO was the only independent protective factor for in-hospital mortality (OR = 0.375, *p *= 0.041, 95% CI 0.146–0.963). Concurrent IABP initiation with ECMO had less need for continuous renal replacement therapy (30.6% vs. 49.3%, *p *= 0.039) and less neurological complications (8.2% vs. 22.7%, *p *= 0.035), but more thrombosis complications (18.4% vs. 2.7%, *p *= 0.007).

**Conclusion:**

Concurrent initiation of IABP with ECMO provides better short-term survival for PCS, with reduced peripheral perfusion complications.

## Background

The incidence of postcardiotomy heart dysfunction is about 3–5% [1], and nearly 1% require circulatory support for postcardiotomy cardiogenic shock (PCS) [2]. Veno-arterial extracorporeal membrane oxygenation (VA-ECMO), as short-term mechanical circulation support, has become the first-line therapy in the setting of cardiogenic shock in the last decade [3, 4]. Unfortunately, the weaning rate of VA-ECMO in PCS subjects remains the lowest among all indications and the in-hospital mortality is over 50% [5, 6]. The risk factors of VA-ECMO use for cardiac arrest after percutaneous coronary intervention were previously identified [7]. However, there was no universally agreed guideline on the indications of VA-ECMO for PCS, and factors that affected mortality of such cases remained unclear. In this study, we described our experience of VA-ECMO use for PCS in Fuwai Hospital and identified the factor that associated with in-hospital mortality.

## Methods

### Study population

We consecutively included 152 adult subjects ( ≥ 18 years) who received veno-arterial VA-ECMO for refractory PCS in Fuwai Hospital from January 1, 2005 to December 31, 2017 in the study. We retrospectively collected clinical variables for all the subjects from their clinical records. Our study complies with the Declaration of Helsinki. The Ethical Committee of the Fuwai Hospital approved this study. As this was a retrospective chart review study, requirement of informed consent was waived.

Primary indication for VA-ECMO was intra-operative circulatory instability during or immediately after weaning from cardiopulmonary bypass when the primary surgery procedure was finished. Secondary indications included delayed PCS for progressive heart failure, post-operative cardiac arrest. The decision and the optimum time to initiate VA-ECMO and IABP support was made by the surgical team, which was independent of the study. The clinical criteria of VA-ECMO institution for PCS were defined as follows: hypotension with systolic arterial pressure < 80 mm Hg and mean arterial pressure (MAP) < 60 mm Hg; signs of renal failure (urinary volume < 20 mL/h), anaerobic metabolism and metabolic acidosis (pH < 7.3, lactate level > 3.0 mmol/L), despite optimized supportive measures such as IABP, inotropes, nitric oxide and phosphodiesterase inhibitors. Hemodynamic criteria were cardiac index (CI) < 30 mL/s/m^2^ and pulmonary capillary wedge pressure ≥ 20 mmHg.

### ECMO management

In our center, the surgical team evaluated the patient to decide when the ECMO was indicated according to inclusion criteria mentioned above. The ECMO circuit consisted of a centrifugal pump console (Bio-Medicus BP-550, Medtronic, Minneapolis, MN, USA, or RotaFlow RF-32, Maquet Cardiopulmonary AG, Hirrlingen, Germany) in conjunction with a microporous membrane oxygenator (Carmeda coating Affinity NT or Maxima PRF Plus, Medtronic) or a polymethylpentene oxygenator with a plasma-tight diffusion membrane (Quadrox D, Maquet Cardiopulmonary AG) with integrated heat exchanger and adapted tube. All components were heparin bonded and connected by the shortest possible tubing system. All patients had peripheral ECMO via the cannulae of the femoral vein and artery. An additional 16Fr intravenous needle casing was inserted distally into the femoral artery to prevent leg ischemia.

The ECMO system was implanted under full heparinization, and activated clotting time (ACT) was kept longer than 300 s. Half of the heparin was antagonized with protamine when full ECMO flow was established, aiming for an ACT of 140–180 s unless there was ongoing coagulopathy with hemorrhage. During the first 24–48 h, ECMO blood flow was adequately adjusted to maintain cardiac index of 40 mL/s/m^2^, with an aim to keep mixed venous oxygen saturation (SvO_2_) around 70%, and mean artery pressure of 60–65 mmHg. The oxygenator was examined twice a day for early detection of thrombus formations. After 48 h, cardiopulmonary recovery was daily assessed by hemodynamic, clinical and echocardiographic measurements to define the optimal time of weaning. Weaning was cautiously begun when SvO_2_ ≥ 70%, hematocrit of 30–35%, absence of bleeding, tamponade or left heart distension, left ventricular ejection fraction (LVEF) ≥ 35% and normal blood lactate levels. Flow rate was reduced stepwise to approximately 1 L/min under continuous monitoring of hemodynamic and respiratory variables. When signs of insufficient perfusion occurred during ECMO weaning, the flow was increased again to full, allowing prolonged ECMO support. When the patients were hemodynamically stable on the minimal ECMO flow with satisfactory recovery of myocardial function evaluated by echocardiograph, patients were weaned off of ECMO support and IABP was retained for further evaluation.

### IABP management

The primary care surgeon made the decision of IABP initiation. IABP (Datascope System 98, Datascope Corporation, Fairfield, NJ or AutoCAT2 wave, Teleflex Incorporated, Hilversum, The Netherlands) was inserted through a femoral sheath and with the tip located near the second rib with a 30 or 40 mL IABP balloon, which was judged according to the patient’s height. The support was initiated at a 1:1 inflation–deflation to cardiac cycle ratio, triggering by the R wave of the electrocardiogram. The weaning criteria of IABP were the systolic blood pressure above 100 mmHg without inotropic agent after removal of ECMO. The support was decreased at a 1:3 inflation–deflation to cardiac cycle ratio when weaning program was initiated, and the patients were weaned off of IABP if the hemodynamic condition was stable.

### Statistical analyses

The normality was tested by Shapiro–Wilk test. Continuous variables with normal distribution were expressed as mean ± standard deviation and compared by Student’s *t* test, while those that were not normally distributed were reported as medians (first and third interquartile range) and compared by Mann–Whitney *U* test. Categorical variables were expressed as percentages and compared by Chi-square test. Logistic regressions were used to analyze the predictors for in-hospital mortality. The multivariate logistic regression was performed to identify independent factors using an “Enter” method. *P* values were two sided, and values < 0.05 were considered statistically significant. SPSS statistical software (version 22, IBM Corp., Armonk, NY) was used for the analyses.

## Results

### Baseline characteristics

Baseline characteristics of all subjects in our study are shown in Table 1. The mean age of the subjects was 49.5 ± 14.1 years, and males accounted for 73.7%. Among all subjects, 57 (37.5%) suffered coronary artery disease with 8 (5%) acute myocardial infarction, 37 (24.3%) cardiomyopathy, 32 (21.1%) valvular heart disease, 23 (15.1%) congenital heart disease (CHD) and 12 (7.9%) aortic disease. The original surgery procedures mainly included 49 (32.2%) heart transplantation, 26 (17%) coronary artery bypass graft (CABG) alone, 18 (11.8%) valvular surgery alone, 10 (6.6%) CABG combined with valvular surgery, 14 (9.2%) CHD surgeries and 12 (7.9%) aortic surgery. VA-ECMO was implanted for primary indication in 85 (55.9%) subjects and secondary indications in 67 (44.1%) subjects. IABP was applied in 77 (50.7%) subjects, among whom IABP was implanted concurrently with VA-ECMO in 49 (32.2%) subjects and sequentially in 28 (18.4%) subjects.

**Table 1:** Baseline characteristics of the patients

	All (*N* = 152)	Survivors (*N* = 73)	Non-survivors (*N* = 79)	*P* value
Age (years)	49.5 ± 14.1	48.1 ± 14.8	50.8 ± 13.5	0.245
Men [*n* (%)]	112 (73.7)	58 (79.5)	54 (68.4)	0.121
Body mass index (kg/m^2^)	23.6 (20.8, 25.9)	23.4 (20.7, 25.7)	23.7 (20.9, 26.6)	0.622
Comorbidities [*n* (%)]				
Hypertension	39 (25.7)	11 (15.1)	28 (35.4)	0.004
Hyperlipidemia	33 (21.7)	11 (15.1)	22 (27.8)	0.056
Diabetes mellitus	20 (13.2)	8 (11.0)	12 (15.2)	0.441
PH	37 (24.3)	21 (28.8)	16 (20.3)	0.222
Atrial fibrillation	43 (28.3)	20 (27.4)	23 (29.1)	0.814
Current smoking [*n* (%)]	20 (13.2)	6 (8.2)	12 (15.2)	0.184
Remote MI [*n* (%)]	27 (17.8)	14 (19.2)	13 (16.5)	0.661
History of cardiac surgery [*n* (%)]	22 (14.5)	12 (16.4)	10 (12.7)	0.508
NYHA [*n* (%)]				0.084
I	28 (18.4)	9 (12.3)	19 (24.1)	
II	29 (19.1)	12 (16.4)	17 (21.5)	
III	62 (40.8)	31 (42.5)	31 (39.2)	
IV	33 (21.7)	21 (28.8)	12 (15.2)	
Surgery procedure [*n* (%)]				
Heart transplantation	49 (32.2)	33 (45.2)	16 (20.3)	0.001
CABG alone	26 (17.1)	8 (11.0)	18 (22.8)	0.053
Valvular surgery alone	18 (11.8)	8 (11.0)	10 (12.7)	0.746
CABG + valvular surgery	10 (6.6)	3 (4.1)	7 (8.9)	0.331
CHD surgery	14 (9.2)	4 (5.5)	10 (12.7)	0.126
Aortic surgery	12 (7.9)	3 (4.1)	9 (11.4)	0.096
Emergency	25 (16.4)	9 (12.3)	16 (20.3)	0.188
Off-pump	10 (6.6)	3 (4.1)	7 (8.9)	0.238
CPB time (min)	284.7 ± 146.1	289.3 ± 166.5	279.5 ± 120.6	0.712
Aortic cross-clamping time (min)	83.0 (59.5, 119.5)	83.0 (60.8, 114.0)	83.0 (50.0, 137.3)	0.901
Preoperative test				
Albumin (g/L)	41.0 (38.4, 45.0)	41.0 (38.4, 45.5)	40.8 (38.2, 44.7)	0.529
Total bilirubin (μmol/L)	24.9 (16.3, 38.5)	27.1 (18.7, 39.8)	23.8 (16.1, 35.3)	0.341
Creatinine (μmol/L)	86.1 (72.7, 105)	87.6 (74.9, 106.2)	84.6 (71.5, 104.5)	0.589
PT (s)	14.0 (13.2, 16.0)	14.1 (13.2, 16.2)	14.0 (13.3, 15.8)	0.882
LDH (IU/L)	214 (174, 288)	221 (178, 313)	207 (172, 275)	0.247
NT-proBNP (μmol/L)	1448 (719, 2909)	1563 (971, 3046)	1234 (508, 2356)	0.110
LVEF (%)	48.6 ± 17.5	43.3 ± 18.6	53.0 ± 15.4	0.001
LVEDD (mm)	56.8 ± 14.1	60.4 ± 15.5	53.8 ± 12.2	0.015
Pre-ECMO implantation [*n* (%)]				
Secondary thoracotomy	45 (29.6)	14 (19.2)	31 (39.2)	0.007
Cardiac arrest/VF	35 (23.0)	8 (11.0)	27 (34.2)	0.001
ECMO implantation [*n* (%)]				
Bedside	41 (27.0)	8 (11.0)	33 (41.8)	< 0.001
IABP implantation	77 (50.7)	37 (50.7)	40 (50.6)	0.995
Concurrent initiation	49 (32.2)	30 (41.1)	19 (24.1)	0.025
IABP first	14 (9.2)	3 (4.1)	11 (13.9)	0.037
ECMO first	14 (9.2)	4 (5.5)	10 (12.7)	0.126
Supporting time (days)				
ECMO	4.8 ± 2.7	5.2 ± 2.2	4.3 ± 3.0	0.047
IABP	6.6 ± 4.2	7.4 ± 2.7	5.9 ± 5.2	0.140

Characteristics of survivors and non-survivors were compared. Continuous variables with normal distribution were compared by Student’s *t* test. Categorical variables were expressed as percentages and compared by Chi-square test

*CABG* coronary artery bypass grafting, *CHD* congenital heart disease, *CPB* cardiopulmonary bypass, *ECMO* extracorporeal membrane oxygenation, *IABP* intra-aortic balloon pump, *LDH* lactic dehydrogenase, *MI* myocardial infarction, *LVEF* left ventricular ejection fraction, *LVEDD* left ventricular end-diastolic diameter, *NYHA* New York Heart Association classification of heart failure, *PH* pulmonary arterial hypertension, *PT* prothrombin time, *VF* ventricular fibrillation

### Outcomes

There were 86 (56.6%) subjects successfully weaned from VA-ECMO, among which 73 (48.0%) survived to discharge. Mean VA-ECMO and IABP support time was 4.8 ± 2.7 days and 6.6 ± 4.2 days, respectively. Characteristics of the survivors and non-survivors were compared as listed in Table 1. Compared to non-survivors, survivors had less hypertension morbidity (15.1% vs. 35.4%, *p *= 0.004), more heart transplantation procedures (45.2% vs. 20.3%, *p *= 0.001), lower rate of secondary thoracotomy before ECMO initiation (19.2% vs. 39.2%, *p *= 0.007) and pre-ECMO cardiac arrest/ventricular fibrillation (11.0% vs. 34.2%, *p *= 0.001). However, we found subjects in survivor group had a lower LVEF (43.3% vs. 53.0, *p *= 0.001) and a larger left ventricular end-diastolic diameter (LVEDD, 60.4 mm vs. 53.8 mm, *p *= 0.015) than those in non-survivor group. Interestingly, we detected much less subjects initiated VA-ECMO by bedside (11.0% vs. 41.8%, *p *< 0.001) among survivors than that among non-survivors. Although both groups had a similar rate of IABP implantation, concurrent initiation of IABP with ECMO was significantly higher in survivors (41.1% vs. 24.1%, *p *= 0.025). Multivariate logistic regression analysis was performed to analyze relevant factors that might affect in-hospital mortality (Table 2). The result indicated concurrent initiation of IABP with VA-ECMO was an independent protective factor for in-hospital mortality (OR = 0.375, *p *= 0.041, 95% CI 0.146–0.963).

**Table 2:** Univariate and multivariate logistic regression

	Univariate analysis			Multivariate analysis		
	OR	95% CI	*P* value	OR	95% CI	*P* value
Hypertension	3.094	1.405–6.817	0.005	2.399	0.778–7.403	0.128
Heart transplantation	0.308	0.150–0.630	0.001	1.159	0.282–4.773	0.838
LVEF	0.968	0.948–0.987	0.001	0.971	0.929–1.015	0.199
LVEDD	1.035	1.006–1.065	0.017	1.001	0.955–1.048	0.981
Secondary thoracotomy	2.722	1.302–5.689	0.008	1.112	0.325–3.812	0.865
Cardiac arrest/VF	4.219	1.769–10.061	0.001	1.402	0.336–5.857	0.643
Bedside	5.829	2.467–13.771	< 0.001	3.051	0.770–12.095	0.112
Concurrent initiation	0.454	0.226–0.910	0.026	0.375	0.146–0.963	0.041
IABP first	3.775	1.009–14.132	0.048	0.963	0.186–4.978	0.964

Logistic regressions were used to analyze the predictors of in-hospital mortality. The multivariate logistic regression was performed to identify independent factors using an “Enter” method

*IABP* intra-aortic balloon pump, *LVEF* left ventricular ejection fraction, *LVEDD* left ventricular end-diastolic diameter, *VF* ventricular fibrillation, *CI* confidence intervals, *OR* odds ratio

### Complications

Complications are shown in Table 3. Renal failure being the most common complications was detected in 68 (44.7%) subjects, followed by neurological complications in 29 (19.1%) subjects. Compared to subjects who received VA-ECMO alone, subjects concurrently received IABP with VA-ECMO had less need for continuous renal replacement therapy (30.6% vs. 49.3%, *p *= 0.039) and neurological complications (8.2% vs. 22.7%, *p *= 0.035), but more thrombosis complications (18.4% vs. 2.7%, *p *= 0.007).

**Table 3:** Complications

Complications [*n* (%)]	All (*N* = 152)	ECMO alone (*N* = 75)	Concurrent initiation (*N* = 49)	*P* value
CRRT	68 (44.7)	37 (49.3)	15 (30.6)	0.039
Neurological complications	29 (19.1)	17 (22.7)	4 (8.2)	0.035
Limb ischemia	25 (16.5)	12 (16.0)	7 (14.3)	0.796
MODS	21 (13.8)	8 (10.7)	6 (12.2)	0.786
Access-site bleeding	15 (9.9)	4 (5.3)	7 (14.3)	0.087
Thrombosis	13 (8.6)	2 (2.7)	9 (18.4)	0.007
Gastrointestinal bleeding	9 (5.9)	3 (4.0)	3 (6.1)	0.590

Complications of patients who had ECMO alone and those who had concurrent initiation of IABP with ECMO were compared. Categorical variables were expressed as percentages and compared by Chi-square test

*CRRT* continuous renal replacement therapy, *MODS* multiple organ dysfunction syndrome

## Discussion

We reported a total of 152 subjects received VA-ECMO for PCS, which was the largest group of subjects in mainland China. The weaning rate and in-hospital mortality were 56.6% and 52.0%, respectively. Multivariate logistic regression indicated concurrent initiation of IABP with VA-ECMO was an independent protective factor for in-hospital mortality. Concurrent initiation of IABP reduced the need for continuous renal replacement therapy and the rate of neurological complications, but increased thrombotic risk.

In such a critical situation as PCS, VA-ECMO is widely used to facilitate myocardial recovery. VA-ECMO supporting in PCS has favorable long-term outcomes, but a high risk of in-hospital mortality [8]. Therefore, short-term outcomes ought to be the key point in quality improvement. As there is no specific guideline for VA-ECMO use in PCS, the decision for initiation of VA-ECMO is mainly made by personal experience of the surgical team. Herein, we summarized our experience in order to improve the outcomes of VA-ECMO for PCS in the future.

The general survival rate of discharge was comparable to previous studies [9]. Different risk factors for ECMO use in PCS, such as age, diabetes, pulmonary hypertension, atrial fibrillation and CABG procedure, were identified in previous studies [10–13]. In univariate analysis, we identified hypertension, LVEDD, secondary thoracotomy before ECMO initiation, pre-ECMO cardiac arrest or ventricular fibrillation, bedside initiation of ECMO and initiation of ECMO after IABP, which represented more terrible conditions, were risk factors for in-hospital mortality, while heart transplantation, lower LVEF and concurrent IABP initiation with ECMO were identified as protective factors for in-hospital mortality. Different from previous studies, which included more CABG or valvular surgeries [9], we had more heart transplant procedures in our study. Additionally, as there were more heart transplantation procedures in survivor group, suggesting a more subjects with end-stage heart failure at baseline in survivor group, we found the mean LVEF was lower in survivor group. In order to eliminate the effect of specific surgical procedure on survival, we applied multivariate logistic regression to identify the independent factor associated with in-hospital mortality. In multivariate analysis, we found concurrent initiation of IABP with VA-ECMO was the only independent protective factor for in-hospital mortality.

It has been controversial for a long time whether additional use of IABP could improve survival or not [14–17]. As previous study included subjects for all indications, confounding factors might be induced when interpreting the yield of combined use of VA-ECMO and IABP. Moreover, previous studies on VA-ECMO in subjects with PCS did not concern about the time sequence for initiating VA-ECMO and IABP. In this study, we took the time sequence of initiation of VA-ECMO and IABP into consideration, so we found concurrent initiation of IABP with VA-ECMO could bring better outcomes. As we know, VA-ECMO increases left ventricular (LV) afterload and decreases the blood flow in coronary artery due to retrograde blood flow, which potentially deteriorates cardiac function. An IABP could reduce LV afterload and increase the blood flow of coronary artery by providing pulsatile flow through inflating during diastole and deflating during systole. Additionally, in such condition as PCS, surgical procedure refined the structural defect of the diseased heart. Reduced LV afterload and increased the blood flow of coronary artery would promote myocardial recovery Therefore, it could improve survival when initiating IABP concurrently with VA-ECMO in such subgroups of patients with PCS.

As pulsatile flow provides better peripheral perfusion, concurrent initiation of IABP with ECMO would improve the blood supply for the kidney and the brain. Therefore, it would be the mechanism for concurrent initiation could reduce the need for continuous renal replacement therapy and decrease neurological complications. However, as additional implantation of IABP increased extracorporeal circuit, which brought extra strike on coagulation system, it put the subjects under more risk of thrombosis. Thus, it would be cautious to monitor blood coagulation and thrombosis in this group of subjects.

In summary, in order to improve the short-term survival and reduce complications related to peripheral perfusion, we suggest concurrent initiation of IABP with VA-ECMO in subjects with PCS if there are no contraindications. Meanwhile, an additional attention should be paid on monitoring coagulation function and potential thrombosis complications.

### Limitations

As a single-center retrospective study, this study is subject to all limitations of a non-randomized study. Our findings need to be further confirmed in a larger multicenter cohort or a randomized controlled trial.

## Conclusions

Concurrent initiation of IABP with VA-ECMO provides a better survival rate to discharge for subjects with PCS and concurrent initiation reduced peripheral perfusion complications.

## Abbreviations

CABG: coronary artery bypass graft
CHD: congenital heart disease
CI: cardiac index
IABP: intra-aortic balloon pumping
LV: left ventricular
LVEDD: left ventricular end-diastolic diameter
LVEF: left ventricular ejection fraction
MAP: mean arterial pressure
PCS: postcardiotomy cardiac shock
SvO_2_: venous oxygen saturation
VA-ECMO: veno-arterial extracorporeal membrane oxygenation
